# Behavioural repertoires in moving crowds: an observational approach

**DOI:** 10.1098/rsos.241561

**Published:** 2025-02-19

**Authors:** Anna Sieben, Tom Postmes

**Affiliations:** ^1^ Forschungszentrum Jülich, Institute for Advanced Simulation (IAS-7), Jülich, Germany; ^2^ Fakultät für Human- und Sozialwissenschaften, Bergische Universität Wuppertal, Wuppertal, Germany; ^3^ Faculty of Behavioral and Social Sciences, University of Groningen, Groningen, The Netherlands

**Keywords:** behavioural repertoire, pedestrian dynamics, crowd psychology, bottleneck, behaviour observation

## Abstract

The wide variety of behaviour found in crowds is a challenge for current models of crowd movement behaviour. To aid the development of a new generation of models, this paper develops a systematic observational approach based on social psychological knowledge about how humans recognize and use social meaning and structures. To develop this approach, we studied the movement behaviour of participants in a pedestrian crowd experiment, more specifically in four experimental runs (*n* = 351) of crowd situations, videotaped from a top-view perspective. In the experiments, large groups of around 80−90 imagined being on the way to a concert. There was no instruction for how to behave except that participants’ motivation to arrive at the gate first was varied through instructions (low/high). Through a qualitative, iterative process of systematic observation a complete list of behavioural repertoires (an ethogram) was collected. Behaviours were performed by either individuals, small interactive groups or large action groups. The observational dataset was enriched with pedestrian trajectory data, used to create heatmaps for density and speed, as well as time–distance plots. The analysis reveals that participants show many, sometimes rapid, changes both between movement repertoires and between the social unit they are engaged in.

## 1. Introduction

The field of pedestrian dynamics seeks to understand and predict the movement of human crowds. Through simulation and experimentation many different collective phenomena have been examined, such as lane formation in bidirectional streams or clogging at bottlenecks as well as complex transport properties like the speed–density relation or bottleneck flow [1,2]. For several decades, a wide variety of models have been developed to describe such behaviour, mainly (but not entirely) based on a physical perspective on crowds as moving particles. However, one challenge for this field (and the models offered) is to deal with and describe the wide variety and changeability of behaviour that are at least partly the result of the complex social psychology of the situation [3]. Crowd behaviour is socially meaningful and thereby linked to social norms, group processes, social identity, communication or social roles [3–7]. Accordingly, in the last decade, social psychology has been more and more integrated into pedestrian or evacuation models [4,5,8]. In particular, Kleinmeier *et al.* [3] have showcased how observations of a variety of pedestrian behaviours in an experiment can be integrated into an agent-based model by incorporating choice via a psychological modelling layer.

The current paper seeks to add to this literature, by closely describing movement behaviour that can be seen in crowds in experimental situations and analysing it as socially meaningful. Social psychological research has shown that humans are prone to make various social inferences when observing (or participating in) social interactions. For example, people readily identify (and to some extent project) social meanings such as intentions and relations both when observing movements of individual actors (even when these are symbolically represented; e.g. [9,10]), and also when observing larger groups and aggregates [11–13]. Indeed, various behavioural attributes make it more likely that perceivers and participants will see collections of humans as a group or an entity: similarity, moving together, common goals and outcomes and social interaction [12,14]. The fact that these inferences are made so readily suggests that they are quite fundamental to humans’ understanding of their social environment. Such processes of inferring togetherness or of shared intentions also operate in groups of people that, together, engage in coordinated movement [15,16]. This demonstrates the importance of such inferences to decisions and behaviours inside crowds, too. Putting all this together, we conclude that if one wants to describe social movement behaviours that we see in crowds, in such a way that it corresponds with the meanings of those actions and interactions inside the crowd itself, it would be a good starting point to attend to the social inferences that we (as humans) make when observing crowds.

From this starting point, the current paper develops a new method for describing crowd behaviour, allowing it to be studied in a more structured and systematic way in the future. This method focuses on observable forms of behaviour and the social meanings and implications of these coordinated actions inferred by observers. In a sense, we combine the objective properties of the behaviour with a more subjective, interpretive layer, in the expectation that these interpretations play an important role both for onlookers and for actors themselves [15,16]. In the following, these socially meaningful forms of behaviour will be called behavioural repertoires.

**Figure 1.**
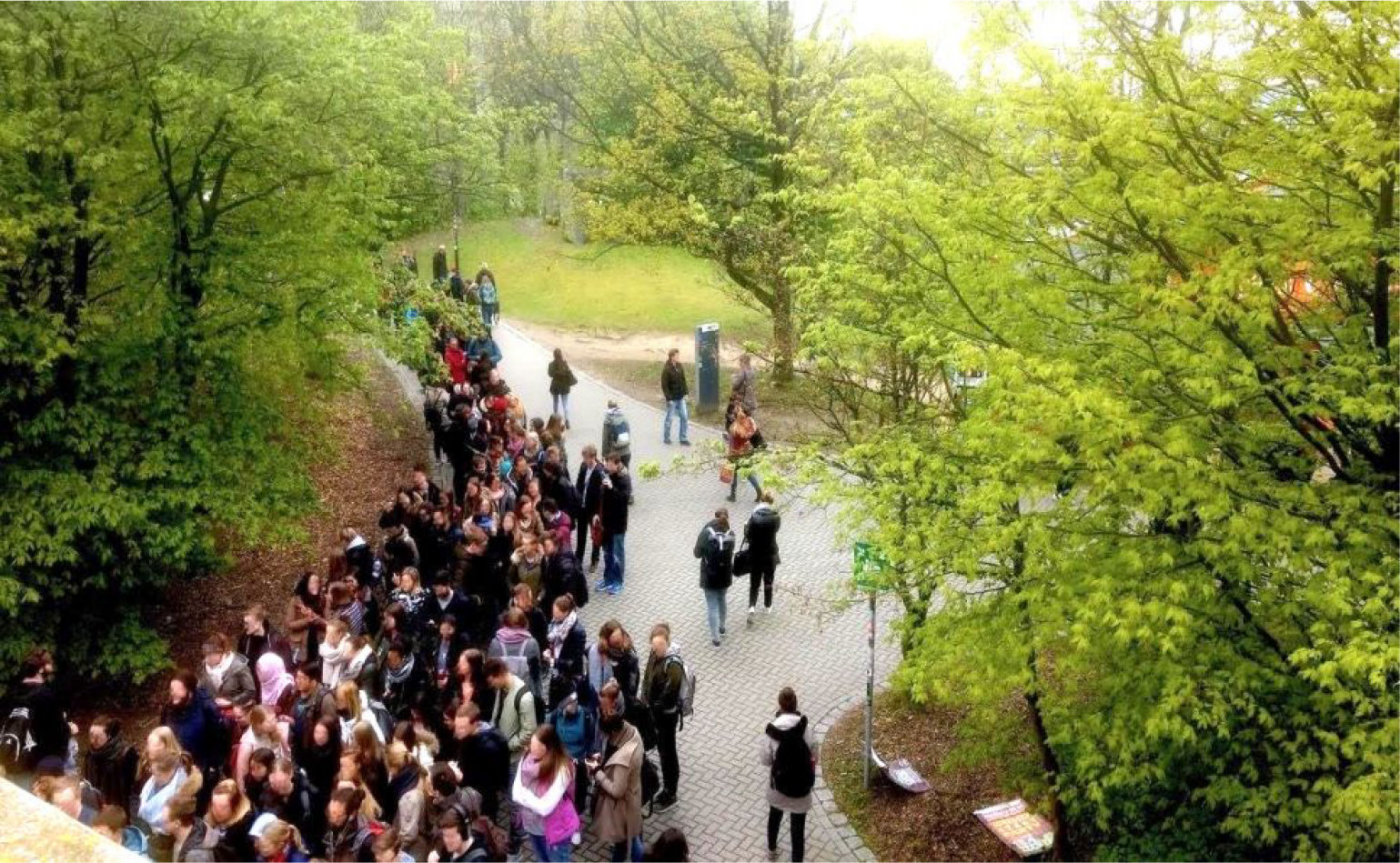
A crowd of students waiting for gift bags (image: authors’ own).

The observational method introduced in this paper allows one to identify discrete forms of behaviour in crowds which are likely to be consequential both psychologically and behaviourally and therefore are likely to influence movement and spatial structures in various ways. An example is shown in figure 1: students gather in front of a distribution point for gift bags. In the front, students form a huddle, approximately eight people wide and nine people deep. In the middle and rear areas, students wait in a queue. The queue is approximately five people wide in the middle area and approximately three people wide at the back. Calling these formations ‘huddle’ and ‘queue’ indicates a way to identify different behavioural repertoires, each with their own social norms. These behavioural repertoires result in different ways of using space. We think that future simulations of collective dynamics can make use of such descriptions in terms of repertoires in order, for example, to better predict the use of space by a queue.

Additionally, in these different forms of behaviour the individual crowd members are, we shall argue, not always acting as independent agents. Instead, alongside already existing social groupings inside the crowd, a multitude of different ad hoc social groupings may emerge organically, through coordinated action (i.e. members of a queue who form a temporary social unit). Our hope is that this way of analysing and describing crowd movement will unlock new pathways to researching the complex variety and structure of pedestrian dynamics. Ultimately, our aim with this is not just to describe the diversity of behaviour in crowds, but to offer an instrument by which this diversity can be incorporated in future models and theories of crowd dynamics.

### 1.1. Studying the movement behaviour of individuals and groups in pedestrian dynamics

In the field of pedestrian dynamics, the traffic flows of humans are traditionally studied from engineering and physics perspectives. From an engineering perspective, a pedestrian’s movement is seen as being the result of choices made concerning the destination, mode of transport, route, etc. [2]. In psychological terms, one might say these are resulting from an actor’s intentions to get somewhere with a specific urgency. Along the route, this original intention may alter or transform as a result of various interactions with their environment, such as the characteristics of the space, the objects one encounters, information one receives or by encounters and interactions with other people (see, for example, an analysis of the dynamic changes of individual motivation in an entrance scenario: [17]).

Formulated in this way, it may seem as if the focus of this field is quite individualistic, but that is certainly not the case: the notion that social interactions will shape and influence how people move in crowds has been long acknowledged [3,18–20]. Interactions with other actors may also provide information for what behaviour is possible or appropriate [21–23]. Many studies have observed the tendency for people to follow others and form lines [24,25]. Also, many studies in this field have devoted attention to the influence of factors such as group size on aspects of movement such as velocity, step frequency and interpersonal distance [20,26–30].

Nevertheless, a recent review by Feng *et al*. [2] notes that the high cost of field and laboratory research ‘limits the amount of research being performed featuring pedestrian operational movement behaviour’. We believe this observation reflects a desire to study more closely how movement is actually executed inside the crowd, including the role of social interactions. Beyond factors such as velocity, directionality and density, can we advance our understanding of how individuals and subgroups move inside the crowd? In the current paper, we argue that there are relatively inexpensive and (we believe) effective ways of doing more with the data that are already available (from experiments, field studies or CCTV) by applying a systematic observation method.

From the current paper’s perspective, what we find noteworthy about the literature discussed above is that the spatial analysis of the movement itself tends to be disconnected from the social and cultural meanings one can attribute to it. We believe that it may be fruitful to reconnect those two into what we call a behavioural repertoire. Two examples can illustrate this: if people in crowds form lines or lanes such as in intersecting traffic flows, or when passing a bottleneck [21,24,25,31] then this may be accidental or it may be seen and interpreted (both by onlookers and by participants) as ‘queueing’. When the social attribution of ‘queue’ is applied to the behaviour, however, then this should change the expectations and interpretations of the behaviour of self and others [7,32,33]. As this illustrates, the analysis is potentially enriched if the social meaning of coordinated behaviours is taken into account. A second example which illustrates this stems from research showing that the (sometimes accidental) performance of coordinated behaviour can lead to a psychological sense of unity and connectedness [16,34–36]. In this way, too, a more detailed analysis of different ways in which unity of action may occur or emerge within crowds may shed light on emergent social groupings, and thereby extend the field’s current focus on pre-existing bonds, relations and identities [2].

We think that the analysis of behavioural repertoires is also relevant for physical approaches in pedestrian dynamics, modelling in particular. A recent review article gives an overview of the different approaches to studying crowds from this perspective, at different scale levels [37]. Most relevant for the current paper is the mesoscopic level of describing movement of large numbers of discrete individual pedestrians. This research develops microscopic agent-based models of self-driven particles and calculates how this affects crowd movement in computer simulations [38,39]. One impulse was the social force model [40] which inspired many researchers and initiated a wide range of model developments (see reviews and literature analysis in [39–41]).

Characteristic of most models is that in them, the intentions of pedestrians are predefined (e.g. the intended velocity is an external parameter). Changes in intentions to act have only rarely been taken into account, with the exception of models of route choice. But even when the route does not change and when there is an overarching intention to ‘move forward’, one can see in practice a wide variety of ways in which it can be executed, both by individuals and by groups: how do actors choose what to do, inside a specific crowd situation? One important exception is [3], where an agent-based model was built that includes the process of choosing between different repertoires. This, we believe, indicates what direction future model development could take. What is necessary to implement such a step, however, is to gain a more comprehensive (and ideally exhaustive) insight into not just what those repertoires are, but also where they originate (e.g. from environmental features, from common knowledge embedded in shared identities or culture) and how they are enacted (either individually, or in smaller or larger groups, or indeed by entire crowds). In order to achieve this, it is necessary to more closely study how the actions of multiple actors are structured or organized in relation to one another. This is the rationale for the current paper.

### 1.2. Current research

To put it simply, the objective of the current paper is to explore an observational approach to examine the heterogeneity of behaviour in experiments that were developed to study pedestrian dynamics from an assumption of it being homogeneous and predictable. The original goal of these experiments was to examine how different geometries, signage and motivations affect participant behaviour, and thus density, speed and flow, before and within an entry gate (resembling the entrance of a concert venue).

The collaborative work that led to the present article was done after the experiments were conducted. The starting point was our observation that on the videos of the experiments more different behaviours and changes between behaviours can be seen than the comparison of experimental conditions can bring to light. In order to capture these, an exploratory approach had to be taken, which was increasingly systematized in an iterative process (as described in [42]). As will become clear further on, the findings do not just shed light on the particular patterns of heterogeneity: they also reveal homogeneities of behaviour and seemingly coordinated changes between homogeneous behaviours, which invited us to develop a theoretical framework to account for what we shall refer to as behavioural repertoires.

To develop our framework of analysis, this paper picks up McPhail’s sociological approach for studying collective and individual actions in gatherings with systematic observation protocols (e.g. [43]). Our analysis, however, focuses more closely on movement behavioural repertoires (whereas McPhail studied primarily political and religious behaviour at large gatherings). Additionally, the observation method was informed by behavioural biology (and the technique of the ethogram) and by recent works in psychology that investigate helping behaviour in public spaces [44]. In biology, a behavioural repertoire is defined as a ‘holistic compilation of behaviours that are observed and then described by an ethologist for a particular animal species. These catalogues are essential to the scientific study of behaviour for purposes of standardisation. Repertoires are designed to be comprehensive’ [45]. Following this definition, the goal of our observation is to produce a complete list of behaviours for the experimental runs analysed.

In three respects, however, our term ‘repertoire’ deviates from its usage in biology. (i) In biology, repertoire is used in the singular as an overview of all possible behaviours. However, we refer to each individual behaviour (to race, to queue) as a repertoire. This is because ‘queueing’ is a human concept which already includes multiple behaviours, such as stopping, lining up, not passing, etc. Similarly, racing can involve running fast, pushing away, preventing overtaking. (ii) Another reason to not use the term behavioural repertoire in its purely biological meaning is that queuing—as an example—draws on a cultural and embodied understanding of what it is to queue (or to race or to march, etc.). This understanding includes scripts, social norms, social structure (see [46] for the queue) and expectancies concerning emotions, atmosphere or communication. Furthermore, these behavioural repertoires are associatively linked to specific cultural and social contexts, e.g. a competition, an intake, a military parade, and these associations enrich the meaning of the repertoire (see [47,48] for a similar use of the concept repertoire). Thereby, it also became clear during analysis that our coding could not just consist of the observing and recording of overt behaviour only. We realized that an interpretative approach was required which ascribes meaning to the observed bodily movement based on a more or less shared set of cultural knowledge [49,50]. Finally, (iii) the behavioural repertoires in a crowd have different ‘actors’: while one can walk slowly or fast alone, it is only possible to race together. Also, a queue necessarily consists of several people. To join someone requires at least two people who belong together. On the contrary, falling behind might be done by an individual but can also be performed by a subgroup. Therefore, behavioural repertoires can be performed by one, several, or many participants in a crowd. It can be assumed that the performance of these joint actions is enabled by particular pre-formed groupings as can be found in a social movement or shared identity (in line with work on both action repertoires [48] as well as with social identity research [51]). But as shall become clear further on in the paper, it appears that in the crowd situations we studied, the predominant forms of collective action were not tied to one group or identity. For this reason, it is an interesting question as to whether the performance of those actions itself, in social units larger than one person, can instil and create a sense of unity and solidarity [16,34,52].

In sum, our analysis aims at identifying behavioural repertoires (e.g. to queue, or to race) which themselves consist of multiple behaviours bound together by cultural understandings and social scripts which express various forms of togetherness. We therefore ask which behavioural repertoires can be described in a crowd entering an infrastructure and whether behavioural repertoires change within an experimental run. Furthermore, our method is used to observe the social units in which behavioural repertoires are performed. We investigate whether and to what extent social units emerge and disintegrate again. It is the combination of observation, interpretation of meaningful behaviour and differentiation between social units performing the behaviour that makes our method of coding a novel contribution to crowd research.

## 2. Material and methods

### 2.1. Data collection

The current paper analysed a selection of runs from an experiment studying crowd behaviour of large groups (85–90 participants) moving towards and through a bottleneck (see [53] for details). The full experiment consisted of 45 experimental runs with 1058 participants (age range 18−85, median 31 years; 51% female, 47% male, 2% non-binary) in total. The experiments took place in Düsseldorf, Germany, in a large multipurpose event hall. Ethical approval was given by the German Psychological Society (DGPs, file reference SiebenAnna2019-10-22VA). Participants were recruited via local media (newspaper, radio), social media and universities. Selection criteria were minimum age of 18 years, body height of 1.5−2 m, robust physical health, ability to walk and stand for hours and mental ability to be part of a large and dense crowd. All participants signed an informed consent form, agreed to being videotaped during the experiments and to footage being published in an open science data repository. Due to COVID-19 restrictions in 2021, everyone was tested for a COVID-19 infection before entering the venue and participants had to wear surgical masks during the experiments. To accustom participants to being in a large group at higher density in times of ‘social distancing’, an ‘icebreaker’ task was carried out first. The entire group was led by the person in charge into a corridor with two doors where they waited until the last participant had entered the corridor. Then the rear door was closed (a density of about 1 participant m^−2^). The person in charge waited for a few minutes before moving into another open space [53]. Participants then entered into the experiments. At the end of the day, participants completed a questionnaire about feelings of safety during the experiments, including fear of COVID-19 infection. Because results showed that participants felt safe, we do not assume the pandemic distorted results [53]. Anecdotally, participants showed enthusiasm throughout the day in experiments with close proximity and high momentum.

On the day of the experiment, the participants were divided into large groups (each with 85−90 people) in such a way that groups of friends were separated. In some cases, however, people who already knew each other participated together. Since each large group participated in experiments the whole day, the people got to know each other during the day and formed new groups.

The experimental procedure simulated the entry of a concert event: large groups of around 85−90 were instructed to enter a concert building, without any instructions on how to behave. The instruction took place in a waiting area, separated by a curtain from the experiment area. After a start signal, they moved into a large holding area in front of a small gate, waited for 1.5 min, and then entered the concert hall one by one, through the opened gate. After entering the gate, they were asked to push a button on a feedback terminal to evaluate their experience in the run. They were then guided to a waiting area where they filled out a post-run questionnaire, containing among other things a manipulation check for motivation. The holding area demarcated by metal grids on the left and right led to the entry gates. There were three entry gates in total, but in the conditions used in this paper only the middle one was opened. The metal grids and entry gates are also used in real-life concerts and festivals to manage crowds, to ensure the ecological validity of the physical space.

Instructions were standardized and delivered via a loudspeaker by one crowd manager who was placed on a ladder to oversee the crowd (always the same person). Participants were asked to imagine going to a concert of their favourite artist. The motivation manipulation was achieved by varying instructions: in the low-motivation condition, they were told that seats were reserved and therefore they were not in a rush. In the high-motivation condition, they were told there were no fixed seats. Only those in the front would get a good view. Others would have to settle for a view of the video screen. After the instruction, the curtain was opened and a start signal by the crowd manager initiated the movement into the experiment area. Because the bottleneck was closed by a second crowd manager at this point, participants gathered in front of it. After approximately 2 min, the first crowd manager used his loudspeakers to announce the opening of the gate. Participants took part in several runs throughout the day, although the participants in the runs selected for the present paper were all different individuals.

The crowd movement in the holding area was filmed with five ceiling-mounted cameras (for technical details see [53, tables 1 and 3, figure 14*a*]). Recordings allowed for trajectory extraction and for linking trajectories with participant IDs and other data, such as questionnaires. For the trajectory extraction, all participants wore dark colours and a green bandana with an Aruco marker. The procedure of trajectory extraction is described in [53] and was performed with PeTrack [54]. Global inspection of crowd movement shows there are three phases: (i) moving into the holding area, (ii) waiting in front of the gate, (iii) entering through the gate.

**Table 1.** Complete list of behavioural repertoires.

**codes for uniformly acting (sub)groups**	**moving**	**lagging behind**. Walking more slowly than the rest, often in an obviously bored manner. The group thereby behaviourally sets themselves apart **walking organically**. Aggregates walk but they do so more or less individually, like in a shopping street. This looks ‘organic’ **walking mechanically**. A group can also walk almost in a choreographed manner, by keeping some kind of formation or using similar motion or expression as they move forward. This looks regimented or controlled (‘mechanical’) one way or another. It is similar to marching **running** **racing**. People are also running, but this time they are trying to be first. This has a different feel to it: they are racing each other **overtaking**. There are different forms of overtaking and this can be carried out by action groups, individuals but also by smallish subgroups. (i) People move toward a static or very slow-moving group (a queue or a flock) and they move into a space or a gap (typically on outside) overtaking part of the queue or flock, typically to get to the front. (ii) People move faster than those around them, for example in a group that is walking there are two people running through the group to the front. (iii) More rarely, there are individuals or small groups who move through a static crowd **inching forward**. A group very slowly walks forward, until they meet an obstacle. This is typically stop-and-go, not a continuous flow. This can also occur in a queue: people move forward while remaining a queue formation **gap filling**. People move forward into a gap between other participants (not just behind others); movement that increases density **zipping**. Occurs in front of a bottleneck when people from different sides take turns in order to walk through one after the other, similar to cars when two lanes merge **surging**. Mainly in front of a gate that opens, everyone moves towards the bottleneck at the same time and thereby increases density
	**standing still or other**	**queueing**. Behaviour following a clear set of rules—overtaking and queue jumping are perceived as illegitimate. Queue can have different shapes and widths, but always comes in an elongated shape of a snake. As more people join the queue, the queue has the tendency to broaden out (from one abreast, to two, to three or four or even more). Queuing can be pre-organized and managed but also be a very spontaneous and almost instant action, which can be performed by groups smoothly, immediately following any kind of motion (including racing) **huddling**. As people join from behind and gather before an entrance or gateway, they have a tendency to huddle around the gateway, forming a (semi)circle with small distances between each other, sometimes even touching or pushing each other. Often, we see huddles develop around a queue or when a queue breaks down as people move forward on the outside **audience behaviour**. A group focusing on a common object and actively attending. For example: a group that listens to announcements or the movement/actions of the crowd managers, or a group that stands around and observes a specific event (such as an accident) **standing around**. A group attending and seemingly waiting for ‘things to happen’, without having an active focus on a specific object or event (as is the case when they are an audience). This can be seen for example in postures such as crossed arms, a bit backward leaning, which actively communicates passivity and is interpreted as a ‘wait and see’ posture. In this waiting mode, people tend to distribute evenly across the space occupied by their group, keeping equidistance **keeping distance**. A group holding back or not joining the rest of the crowd. The group thereby behaviourally sets themselves apart. Often with an attitude of boredom
**codes for small groups**	**moving**	**lagging behind**. Walking more slowly than the rest, often in an obviously bored manner. The interactive group thereby behaviourally sets themselves apart **walking**. For the small groups we do not distinguish organic and mechanical walking **joining someone**. Moving in the direction of the entrance in order to wait together with someone they seemingly know **walking behind or next to each other**. Touching shoulders or holding hands in order to stay connected **marching in pair**. Walking together and in lockstep. This is similar to the mechanical walking of large action groups. It looks restricted and regimented **running** **overtaking**. See above **pushing**. A group is using arms, shoulders, upper body to make others move forward or to push others to the side to overtake **gap filling**. See above **letting go first**. Making someone else go first by gesturing, body language or pausing in the movement **inching forward**. See above
	**standing still or other**	**chatting**. Talking with other participants **helping**. This includes different forms of helping, in particular handing back head marker, helping someone up, checking whether someone is fine after a turbulent situation **touching**. Touching someone’s shoulder after joining someone, or holding someone’s shoulder to stay connected **gesturing (e.g. pointing, waving**). Social interaction by nonverbal means
**codes for individual behaviour**	**moving**	**lagging behind**. See above **walking**. See above **running**. See above **overtaking**. See above **gap filling**. See above **inching forward**. See above **letting go first**. See above **pushing**. See above **walking backwards**. Turning around and walking away from the gate
	**standing still or other**	**disengaging**. Demonstrating low motivation and boredom, for example leaning against fence, stepping to the side **picking up or attaching head marker**. Code that is idiosyncratic for the experiment, because participants were wearing head markers that sometimes detached **falling, stumbling** **checking phone**

In total, 45 experimental runs were conducted, which systematically varied motivation to get to the gate first (low/high), geometry of the holding area (straight, bend, width), instruction to act selfishly (no/5%/15%/30%), signs (no/lines/signs), interruption (no/with), and the number of entrances (1/3). For the current analysis, we selected conditions in which behaviour was relatively natural and unconstrained. Accordingly, we deselected conditions with strong spatial constraints or direct behavioural instructions. As training material for the interpretive observational analyses, which were qualitative and iterative, we selected and analysed three runs with a straight or bend geometry, just one entrance, no signs and no behavioural instructions (see below for details). When we were satisfied with the ethogram, we selected four different target runs (total *n* = 351) for structured and independent coding by two raters. These four runs were the most unconstrained and ‘natural’ settings in which people could determine their own actions and movements with little restriction and interference. They had a straight and large geometry, just one entrance, no signs and no behavioural instructions. In order to maximize variability of behaviour encountered, we selected runs with low (experiment IDs 2C020 and 2C070; *n* = 88, 90) and high motivation (experiment IDs 1C060 and 2C120; *n* = 85, 88). As can be seen from §3 and the electronic supplementary material, these four runs contain a wide range of different behaviours that change dynamically. Manual coding is therefore very time-consuming. This is another reason for the selection of four experimental runs. For technical reasons, the demographics of individual subsamples cannot be described, but there is no reason to assume that these deviated systematically from the entire sample.

### 2.2. Analysis of pedestrian dynamics: trajectories, density and speed

**Figure 2.**
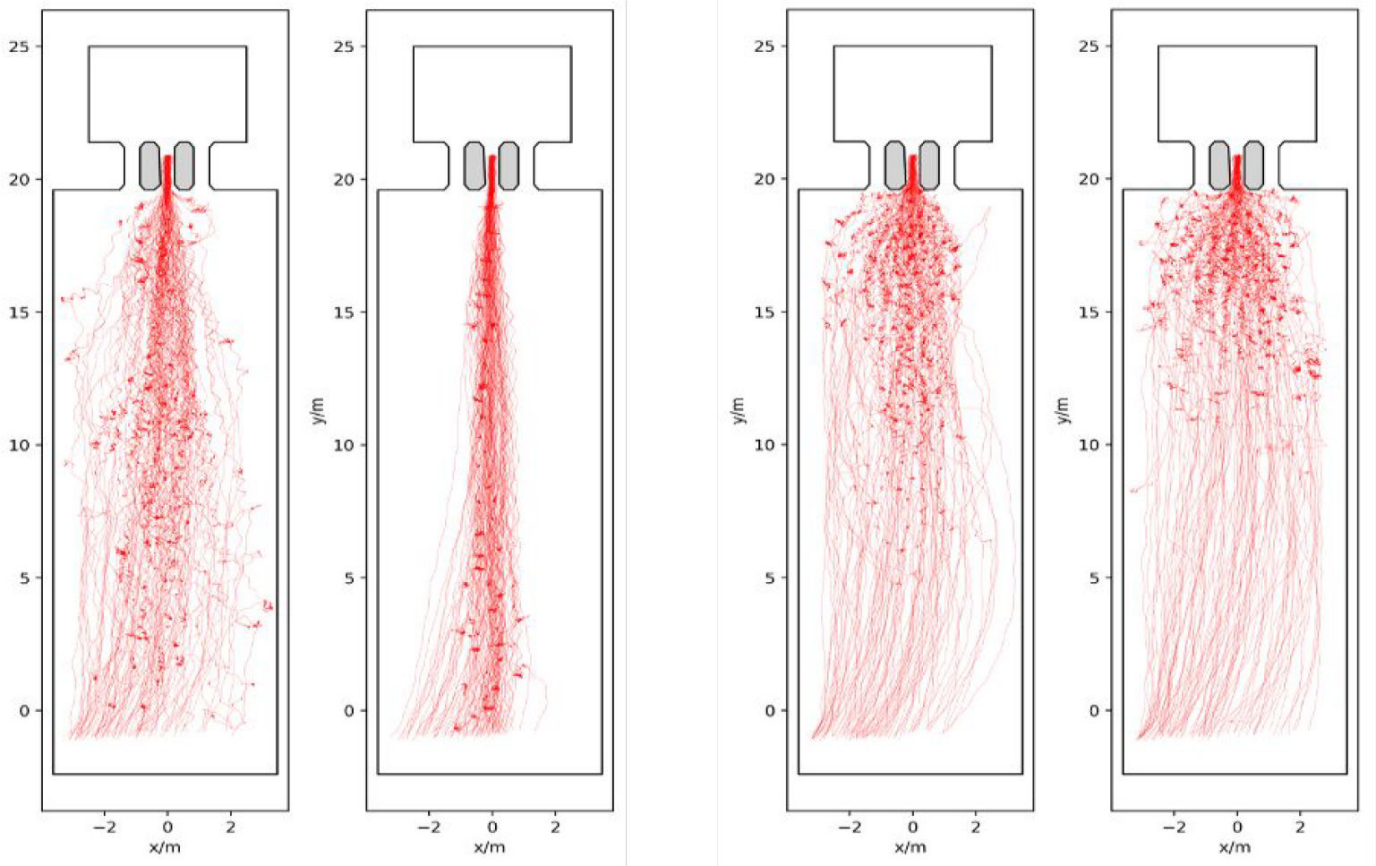
Trajectories of participants and ‘knots’ where they stood still in the holding area, for the low-motivation runs (left) and high-motivation runs (right).

**Figure 3.**
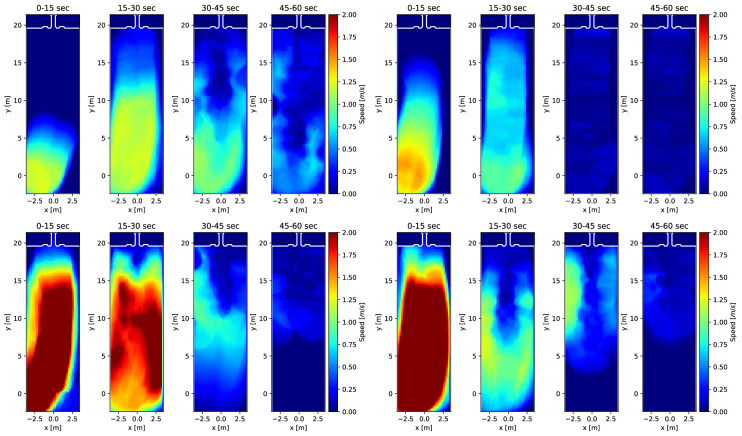
Heatmaps for speed during the entry phase covering the first minute of the experiment, divided into 4 segments of 15 s, for the low-motivation runs (top; left 1C060, right 2C020) and high-motivation runs (bottom; left 2C070, right 2C120).

**Figure 4.**
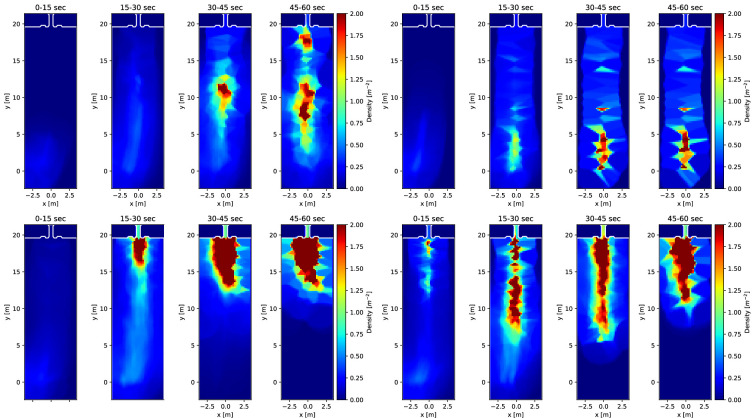
Heatmaps for density during the entry phase covering the first minute of the experiment, divided into 4 segments of 15 s, for the low-motivation runs (top; left 1C060, right 2C020) and high-motivation runs (bottom; left 2C070, right 2C120).

**Figure 5.**
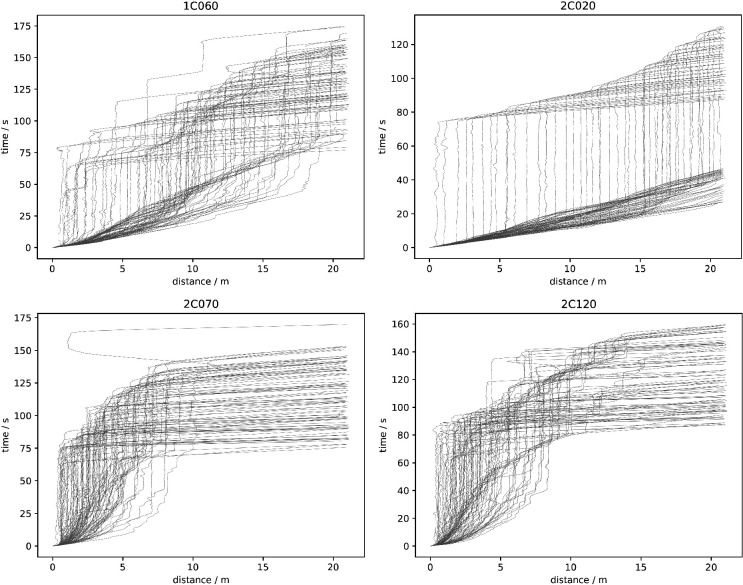
Time–distance plots for the low-motivation runs (top) and the high-motivation runs (bottom).

Knowing individuals’ location in space and time enables various analyses of crowd movement applying methods commonly used in the interdisciplinary field of pedestrian dynamics. The four experimental runs are described in terms of trajectories, density and velocity. This serves to illustrate the movements of individuals in the crowd across the four runs analysed in this paper. The trajectories are visualized on a map of the holding area over the entire time of the experiment (figure 2). Trajectories are based on head movement and participants show slight head movement when standing still, creating ‘knots’ in the location where this occurred. Density and speed are depicted in heatmaps (figures 3 and 4). Speed ranges from 0 to 2 m s^−1^, density ranges from 0 to 2 persons m^−2^. The heatmaps were created by following the procedures described in [55] using the Python library PedPy [56]. The analysis focuses on the time before the entry gate was opened (approximately 1 min), divided into intervals of 15 s. To complete the analysis of movement, we also provide a time–distance plot of the trajectory data (figure 5). In addition to these descriptions, we recommend watching the videos of the experiments, included as a supplement to the paper (https://fz-juelich.sciebo.de/s/LmEXPWzJu4SyLbn).

### 2.3. Development of ethogram

The primary purpose of this analysis was to identify different behavioural repertoires by systematically observing behaviour in the holding area. We adopted a method used in behavioural biology to inventorize behaviours of an animal in a certain habitat as completely as possible [45]. Such an ethogram is essentially an elaborate and structured list of all observed behaviours. It is developed in an iterative qualitative procedure in which observed behaviours are noted, described, interpreted and categorized. As the later developed extensive ethogram shows, this approach aims to capture behaviour as completely as possible. For this reason, the behaviours they describe are not always mutually exclusive, making an ethogram different from a coding scheme.

The ethogram was developed on three training runs, using a pre-registered inductive procedure in an iterative process of months (see https://doi.org/10.17605/OSF.IO/HSTX8). In the first stage, the authors watched one video of the training runs (experiment ID 3C090) together, shared observations and discussed interpretations and implications. Over the first stage of coding, consensus was reached on some of the key characteristics of the analytic approach, including for example the ‘discovery’ that interpretive observation allowed for the identification of meaningful behavioural structures performed by large groups or interactive groups. In this initial stage, we decided that because there were between-run differences, our ethogram could not be a ‘closed’ system: new categories could be introduced at all stages. Still, as the process went on, fewer and fewer ‘new’ behaviours were added. Moreover, because the ethogram had to be developed from a bird’s eye view where certain behaviours are very well visible (e.g. movements of arms and legs) and others less so (e.g. facial expressions), we decided not to include associated behaviours (e.g. laughing, pointing out) in our ethogram. Instead, we recorded behaviours that were clearly and easily recognizable on the available video material.

In a second iterative stage, the authors created a first version of the list of behaviours. They now watched the first training video alone, using the existing list to annotate the behaviours observed but also extending the ethogram when needed. In a third stage, they used other training runs to develop the procedure of coding (experiment ID 3C100), and one run to code and to calculate inter-rater reliability (experiment ID 3C120).

At stage three of the coding, we realized that group actions can be identified reliably only when there is motion. We therefore marked stills as being from the dynamic or static phases and coded action-based groups on the dynamic ones only. Also, because small group interaction was observed mainly when groups were static (queuing, waiting, huddling), most of those codes were assigned on the static stills. Finally, individual distinctive behaviour was found in both dynamic and static phases.

The material for the final structured observational analysis (stage 4) consisted of videos of the four experimental runs from the top-view perspective. We selected a camera overseeing the entire area [53]. Videos were between 3 and 4 min long. We coded the movements on the basis of watching the video itself. However, codes were recorded by annotating stills (embedded in slides). For each video, 12 stills were selected. In the three phases of the experiment a different amount of activity takes place; therefore in the first phase (moving into holding area) seven stills were taken, with a 5 s interval between the first four and then a 10 s interval (the reason for which was the high level of activity in those first 20 s). In the second phase (waiting in front of gate), we selected two stills and in the third phase (moving out) three stills.

**Figure 6.**
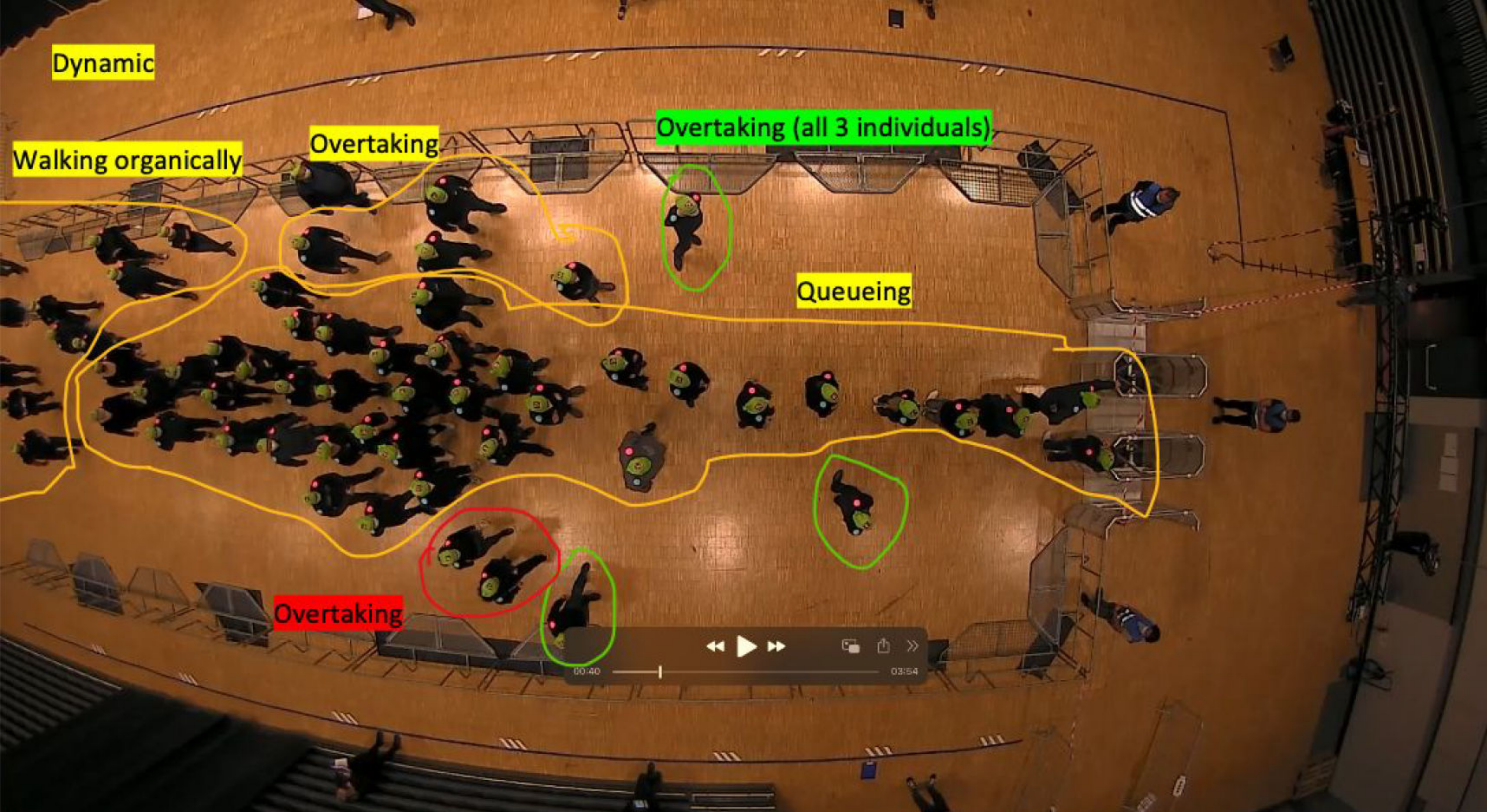
Exemplary screenshot (low-motivation run 1C060, at 40 s) showing how different social units (green: individuals, red: small interactive groups, yellow: large action groups) and their behavioural repertoire are coded.

The final coding of data was structured in two steps. First, coders (both authors) independently circled the social units (see figure 6). Larger subgroups displaying coordinated actions were marked in a different colour (yellow), from small groups (red), or individuals (green). In a second step, they used the social unit coding from one set of slides and annotated the behaviour from the ethogram (table 1). Almost all behaviours observed during the ethogram development^1^ were also seen in the final analysis of the four runs reported below.

After coding social units and behavioural repertoires in the stills, we combined this information with participants’ IDs using the PeTrack software [54] which identifies individual participants in space and time. This allows ethogram codes to be linked to physical movement and questionnaire data. Additionally, it transforms the information in the screenshots to codes in a table (via the participant ID). Stills, the coding tables and the video material are part of the electronic supplementary material.

The coding procedure allows us to calculate inter-rater reliabilities (Cohen’s Kappa) and agreement rates at each step of the coding: identification of social units, and of behavioural repertoires. For the social units, the reliability was 0.83 overall (‘almost perfect’ according to [57]) at 85% agreement. Reliabilities for each video ranged between 0.80 and 0.89. Further analysis reveals that the identification of social units was higher for action groups (*k* = 0.85) than for interactive groups (*k* = 0.51, moderate) and individual actions (*k* = 0.65, substantial). For the 182 behavioural repertoire labels, the inter-rater reliability was .84 (almost perfect) at 85% agreement.

### 2.4. Alluvial diagram

In order to visualize the coding results, we made alluvial diagrams of the dynamic phase of the experiment (the first 50 s). These are flow diagrams that can show changes in structure of networks and groups over time. Accordingly, we can illustrate how participants ‘flow’ from one behaviour to another, in different social configurations.

## 3. Results

The results section consists of three parts: first, the physical movement characteristics trajectories, density and speed are described (figures 2–5); second, the ethogram is presented; third, the transitions of behavioural repertoires are analysed and visualized.

### 3.1. Density and speed

Figure 2 shows the trajectories and waiting points for each individual, per run. The analyses reveal differences between conditions and within. As can be seen, the low- and high-motivation runs differ in how space is used: in the low-motivation runs, not the entire width of the holding area is used and participants wait in the entire space. In the high-motivation runs, almost the entire width is used and participants wait much closer to the gate. These differences are particularly evident in the last third before the gate. The images also show differences within conditions: the two low-motivation conditions differ in the width of the holding area used. 2C020 is particularly narrow due to queueing, whereas in 1C060 some individuals use the space on the left and right of the queue. In the high-motivation runs, there are also differences in the width of the holding area used. In 2C070, participants use most of the width symmetrically and they wait exclusively in the upper half. In 2C120, on the other hand, the left side of the holding area is used more than the right. A small number of participants wait further from the gate, in the lower half of the figure.

The temporal and spatial dynamics of the experimental runs can be seen on the heatmaps (figures 3 and 4). In the high-motivation runs, we see much higher speeds and larger areas with a high density. In all runs, speed is highest at the beginning during the first 0−30 s and then gradually slows down. If there is movement beyond that time, it tends to happen around the centre of the holding area where people wait and queue, with individuals moving on the left or right side around them. The counterpart to this movement can be seen on the density maps, where densities are higher in the centre of the holding area where participants are waiting. In the low-motivation runs, a narrow queue can be recognized, while in the high-motivation runs a huddle is formed in front of the gate. As above, there are differences between each experimental run worth noting. For example, in the high-motivation run 2C070, a huddle appears already during the second time interval. In 2C120, huddling is visible only during the last time interval. In the low-motivation runs, we also see differences in density: 1C060 density is higher than in 2C020 and the areas with high density are closer to the entrance gate than in the other run.

Finally, figure 5 depicts the time–distance relationship for each participant. This visualization is a method of illustrating the ‘orderliness’ of the procedure: the almost perfect queue in 2C020 results in a distinctive pattern between time and distance—the orderly structure reveals one participant moving after the other. In all other runs, there is more heterogeneity of movement towards the gate because there are more individual differences in the time–distance relationship: overtaking results in faster progress for some and slower progress for others.

In sum, there are substantial differences between the four runs. Some are due to the condition: high-motivation runs are faster and have higher densities. This is consistent with previous experiments with similar setups and instructions [58]. But the runs also have many idiosyncratic characteristics, ranging from a slow and neat queue (2C020) to a race-like dash to the gate with much huddling (2C070) with much in between, which can only be interpreted by closely observing the nature of actions. This patterned variability of behaviours, as we shall see in the content coding, is a prime reason for the more interpretative development of ethograms.

### 3.2. Ethogram

The physical analyses revealed considerable between-condition and between-run differences. To what extent can these be related to patterns of behaviour? We developed an ethogram to establish this. Most ethograms are developed for coding individual behaviour, but as described above, we code three different social units executing behavioural repertoires: individuals performing distinctive individual actions, small groups (2–4 persons) performing interactive behaviours and larger groups carrying out coordinated and essentially similar behaviours. The ethogram therefore contains different categories of behaviours (see table 1) performed by three kinds of social units. Thus, the question of social units is answered in this study by behavioural observations. This methodological approach differs from other studies that ask questions about social belongingness via subjective data, mostly questionnaires [59]. In the context of moving crowds, however, the situation is quite fluid and we see many different social formations and groupings at the behavioural level even over the course of 4 min which a retrospectively issued questionnaire cannot capture. Additionally, the ethogram distinguishes between movement repertoires and behaviours performed while standing still and/or behaviours that are observed in addition to moving (such as helping behaviour).

### 3.3. Transitions of behavioural repertoires

**Figure 7.**
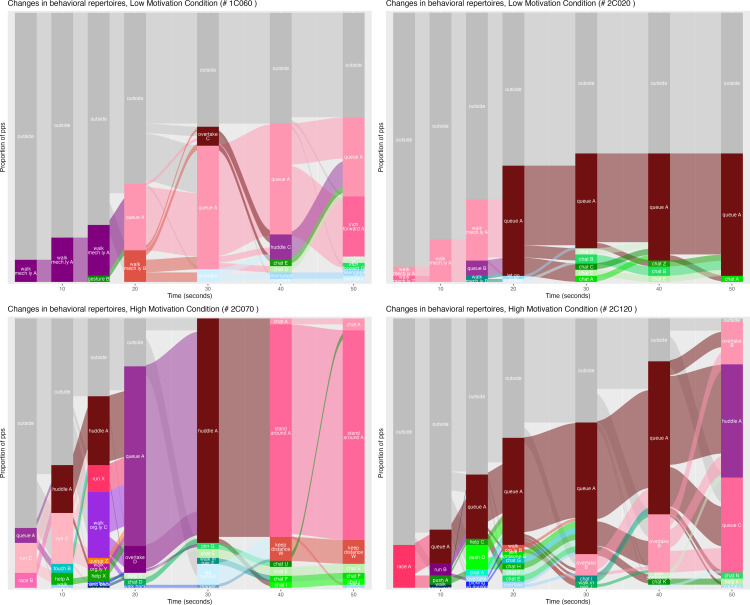
Alluvial charts for two low-motivation runs (top) and two high-motivation runs (bottom). Reddish colours: large action groups, greenish colours: small interactive groups, blueish colours: individuals.

The coded behavioural repertoires as well as their transitions are visualized in figure 7. We want to highlight four results from this analysis that are highly consequential for different aspects of the study of crowd movement.

First, a uniformly acting crowd is rare. In our experiments, this is only visible in the first seconds of the run (uniformly walking or racing) and in one of the runs at the end (everyone queuing). In all other phases of the experiment, several behavioural repertoires can be observed at the same time. Nevertheless, in all runs we see large subgroups displaying relatively uniform behaviours (and these could be identified by observers with very high reliability). From this observation, we conclude that models and theories of crowd movement should allow for homogeneity (especially at the level of subgroups within the crowd) as well as heterogeneity. The relationship between homogeneity and heterogeneity probably depends on the context and the instructions. For example, heterogeneity can be reduced by giving instructions on how to move (‘please run to the exit’).

Second, in low- and high-motivation runs (but more in the high-motivation runs) we see transitions between queuing and overtaking plus huddling. In the high-motivation run 2C120 more and more people join in overtaking whereas in the low-motivation run 1C060 overtaking only happens at one moment in time. In general, more transitions are observed in the high-motivation runs. The low-motivation runs seem to be more structured. In 2C120, people change from racing or running to queuing. This strikes us as a strong change in the repertoire. Whereas racing and running are following the principle ‘the stronger wins’ and allow for overtaking and pushing, the queue is structured by a clear norm not to overtake. In the same run, the overtaking is not initiated by the participants who were racing at the beginning but rather by participants who walk into the experiment structure later. Their overtaking is followed by an interesting dynamic: some of the overtakers join the queue, and at the same time some of the queuers join the overtaking. However, there is a tendency for more and more people to join the overtaking. In sum, we see in the runs many alterations between high degrees of social structure (as in a queue) alternating with the breakdown of structure (as in overtaking). We conclude from this that for theories and models of crowd movement, transitions are key moments to explain. A further analysis of these transitions is intriguing but beyond the scope of this paper as it requires a new method, one that allows to interpret the interactive sequences. We also believe that analysing transitions needs to take the perspective of the participants into account in order to reconstruct what they perceive and to what they react.

Third, with the exception of rare moments at the start of the runs where all move in the same direction (either walking mechanically, running or racing), there are always a few individuals at every moment who separate themselves from the group and who behaviourally individuate themselves by acting differently. The fact that such deviance is rare and can be reasonably well detected (as demonstrated by the substantial inter-rater reliability) shows us how common behavioural unity is, notwithstanding the many behavioural changes within the runs.

Finally, over the course of the experiment, more small groups become visible. We believe this is because individuals within the crowd start interacting more once they have stopped moving as a group. This happens in both conditions, but on top there is more interaction in dyads and small groups in the high- than in the low-motivation runs. This could be because in the high-motivation runs there is more activity (running, queueing, overtaking) and they are more exciting: therefore, there is more to talk about. We think that some of those interactions take place in acquaintances or small groups that may already have existed before the experiment (see above), but that others emerged spontaneously (and sometimes only for a very short time).

## 4. Discussion

This paper describes the development of an observational method and ethogram, on the basis of the social inferences that observers make about the movement of individuals in a crowd. We know that observers attach social meanings to movements of individual actors and groups (e.g. [11,13]). The present study builds on this literature by contributing a methodological and conceptual framework for understanding the variety of movement forms seen in crowds. We shall discuss the contributions made to methods and concepts, the nature of the findings themselves and the theoretical implications of both to the field. At the end of the discussion, we reflect on the limitations.

### 4.1. Methodological and conceptual contribution

The current paper offers a methodological innovation: a method for cataloguing and measuring human behaviour in crowds. Our analytical framework introduces new principles for analysing movement in human social groups. We believe these principles can benefit other researchers, when for example collecting or interpreting automated analysis of human movement. The first principle is that a repertoire makes movements in crowds consequential, both for actors and for observers. For example, compared with a coincidental line of pedestrians, a queue comes with understanding of how to move, and also with norms, expectations and emotions. The behavioural repertoire of queuing thus turns some movements into social gestures: someone who walks past the queue could be overtaking. The implication is that in order to understand how crowds move, it helps to know if they perform a repertoire. The second principle is that in crowds, the shared knowledge of repertoires can self-organize movements. To continue our example, the queue is a product of intentional collaboration which is achieved through a sequence of movements (e.g. slowing down, standing still behind precursors, moving forward when they do, etc.). For the purpose of understanding social behaviour, therefore, the social repertoire is the best level at which to encode actions.

A third principle is that behavioural repertoires in crowds can have different ‘actors’. Repertoires such as queueing or racing are by necessity collective behaviours. Various repertoires, such as walking or falling behind, can be performed individually, in small groups or larger collectives. Thus, identifying repertoires helps one to identify pre-existing as well as emergent social groupings in the crowd. We show that it is possible for observers of crowd behaviour to reach a quite high degree of agreement when coding a new set of crowd experimental data. Thus, the social inferences made by the coders were consistent and predictable.

### 4.2. Empirical findings

Although generalizability still needs to be established, because findings are so far based on a small number of runs of a crowd experiment, the development of ethogram and coding already shows some intriguing and striking results. Future research will refine these findings, but for the purpose of the current paper it is already worth highlighting some findings that speak to the *utility* of the method for theory and future research.

The first finding is that within and across conditions of this experiment in which participants move towards and gather in front of an entrance gate, we observe multiple behavioural repertoires. These behaviours are improvised by the crowd, in the sense that no behavioural instructions were given. Although the experiments lasted only a few minutes, very different behaviours occur in succession and also in parallel. Some behaviours are performed relatively homogeneously by large groups or even the entire crowd, such as walking, queueing and huddling in front of the gate. Some of these behaviours, such as huddling, may require minimal coordination and may even display a breakdown of social coordination, with huddling often occurring after queues break down because of individual or small group overtaking. But other behaviours, such as queueing, require considerable social coordination. It is particularly striking that queueing happened even in the high-motivation conditions, after those at the front of the crowd were racing or running to reach the gate. This means that some groups make an extremely fast and coordinated behavioural transition from racing to forming a queue.

During the iterative research and coding process we tried to find explanations for how this (sometimes very rapid) coordination can take place without there being any behavioural instruction or cues by the experimenter. Particularly noteworthy in this respect was that overt cues to behaviour inside the crowd (e.g. pointing, gesturing) were rare, too. We concluded that the common explanations provided in the literature for such uniformity are implausible. Convergence, for example, cannot explain the rapid transition from one behaviour to another. Emergent norms [60] are not a plausible explanation because at no point in any of our coding did we see in these crowds the coordinating activities (keynoting, milling) by which such emergent norms would be produced. Moreover, in some of the conditions we observed, the changes of behaviour were not emergent but more or less instantaneous and could occur without any deliberation or gestures (e.g. the transition from racing to queuing mentioned above). Finally, it also seemed implausible to us that pre-existing shared identities could explain such rapid changes in behaviour. For one, it is not clear how a shared identity could explain changeability of behaviour at this level: identities tend to prescribe more abstract level intentions. Moreover, it is unclear what this identity might have been and (could one identify one, such as ‘participants’) they are not associated with clear behavioural norms at this level. So the conclusion we reached is that it is most likely that these collective behaviours are grounded in shared cultural understanding of activities such as queueing, racing, marching. This is embodied common cultural knowledge, which participants can mobilize at a moment’s notice, hence our use of the word ‘repertoire’ (cf. [46–48]).

A second result worth highlighting is that we found behavioural repertoires can be performed by different social units: large groups, smaller interactive groups (typically dyads and only occasionally triads or larger units) and individuals. Across the runs, we saw the same pattern: larger social units acting in unison can be identified most easily and reliably when there is motion. Smaller units become more apparent when the crowd is stationary and people are waiting. This was as predicted. What this means is that in the initial phase, when the crowd moves toward the gate, they do so as one or more large groups. As they reach the gate and begin to queue or huddle, this group then divides into smaller interactive groups and it becomes more likely that individuals split off. As the gate opens and the crowd moves through, the individuals may reunite.

In the low-motivation conditions, what is noteworthy is that there is more orderliness and unity of behaviour, with the vast majority (>80%) first walking, then queueing. More interpretively we perceive the manner in which the walking is conducted, in this condition, looks choreographed: the homogeneity of movement is striking and the movement suggests restraint, resembling what we described as mechanical coordination elsewhere (see [16,36]). The idea of mechanical coordination suggests that the walking was coordinated from within the crowd according to a higher order common understanding. This could explain why it looked as if crowd members invested effort in walking in an unnaturally similar way (e.g. in terms of pace, trajectory, motion of limbs, etc.).

More and more rapid transitions are observed in the high-motivation runs. As predicted, more social interactions in small groups happen in the high-motivation runs after a phase of moving quickly: the high energy phase, in which movement tends to be fast, is closely followed by what seems to us like a heightened need to socialize (possibly to share appreciations of what just happened).

### 4.3. Theoretical implications and questions

Even though findings are so far based on a small number of runs of a crowd experiment, they already have theoretical implications. The results clearly show that the assumption of homogeneity in the crowd—both in behaviour and in social units—as formulated by Le Bon [61] does not fit our data. Furthermore, an interesting paradox in comparison with Le Bon’s descriptions appears: in the low-motivation condition, we see more structure and uniformity, but in the high-motivation condition we see collective action that reminds us of Le Bon’s description of crowds as uncontrolled and more emotional, but notably without this affecting the entire crowd but only the homogeneously acting subgroups who are racing, pushing, etc. The latter behaviour can be seemingly without deliberation, more vehement and changeable. Here more behavioural repertoires manifest inside the crowd, hence more within-crowd variability and over-time variability. Therefore, the more uniform acting condition (low motivation) was the least vehement and changeable. The more vehement and crowd-like (high motivation) is on closer inspection also more heterogeneous between subgroups and over time.

Our analysis furthermore resonates with the work of McPhail and colleagues when they describe the dynamics within crowds in the following way: ‘The most characteristic feature of any temporary gathering is the ongoing alternation between individual and collective actions. Individuals interact with their companions and then act alone, they may then act collectively with a larger number of others in the gathering, then interact with their companions, and again act alone’ [47]. And they argue: ‘When people do act together, they are more likely to do so in small numbers, in disparate patches, and often in different ways, across the space and time dimensions of the gathering. When mutually inclusive behaviour does occur on the part of most or all members of the gathering, it is seldom very complex and is never continuous. Instead, gatherings consist of alternating and varied sequences of individual and collective action, sequences that vary from very simplistic to quite complex, for example, clustering, queuing, forming arcs and rings, cheering, applauding and booing [...]’ [47]. The conceptual and empirical question which arises from both our analyses and McPhail and Tucker’s descriptions is how the transitions are coordinated in a crowd: what do participants in a crowd perceive that makes them change their behavioural repertoire? How do they communicate about the appropriateness of a repertoire? In the experiments that we observed the processes of milling and keynoting which have been described in emergent norm theory [60] never occurred. At this point, we see the need for further research that looks at processes of coordination and communication in moving crowds, incorporating the special circumstances, e.g. little time to negotiate, no opportunity to talk to everyone at once. McPhail and Tucker have, indeed, introduced an interesting distinction between three different ways of initiating collective action (as they call it) or behavioural repertoires (as we call it): first, coordination can happen independently because each individual knows a repertoire and behaves accordingly; second, coordination is initiated interdependently after everyone realizes that they need to cooperate to fulfil a task; and third, a leader of some sort gives instructions.

Another conceptual and theoretical question is psychological unity: when does an aggregate or assembly of individuals transform into a psychological crowd and are the observed social units also perceived as such by the participants themselves? The theoretical backdrop to this question is more than a century old. Le Bon formulated the ‘law of mental unity of the crowd’ and portrayed a crowd as having a group mind. This position was fiercely criticized by some (e.g. [62,63]). In modern crowd theories, Le Bon’s ideas are long gone, but the old debate continues. Reicher & Drury [64], for example, talk about the formation of a psychological crowd in their ESIM model. In this model, a crowd forms because of an inter-group dynamic. The experiments analysed in this paper, however, occur in the absence of any obvious inter-group dynamic: there is no real outgroup. This raises the question as to whether in the absence of a psychological outgroup, the psychological motives combined with the intragroup dynamic give rise to the emergence of phenomena that we can associate with the formation of a psychological crowd (which include feelings of unity or displays of unity and/or uniformity). Relevant to this investigation is also the IMIF [16,52] and the work on collective or shared intentionality (e.g. [65]). This work provides pointers to how observing others’ motion and movement are sufficient to induce a sense of common direction and inferred purpose. The implicitness of such hypothetical processes sits well with the environment of the experiments, in which deliberation and exchange were not really possible.

### 4.4. Limitations

We have already cautioned that the current analysis is based (i) on a small set of data, in the form of seven relatively brief runs of on average 4 min and (ii) on a very specific crowd experiment. Accordingly, the generalizability of the ethogram is limited to this situation, for now. We believe it to be robust in this context, because when we looked at new runs we did not add any new repertoires anymore. However, we noticed that when particular events or disturbances occur (e.g. someone falling) we see new repertoires. Accordingly, we expect our ethogram to be robust for the situations which are most common. The generalizability of the ethogram to other crowd situations should be established. It would be reasonable to expect it to generalize to other entrance situations, but we note that there are substantial differences between these experimental and real-life conditions (e.g. a greater diversity of actors, goals, modes of transport, etc.). That said, the number of behavioural repertoires that apply to such situations is limited culturally. In all, we believe the current ethogram is a good start.

Another important limitation to this analysis comes from the perspective of observation: by using only bird’s eye view videos, much information remains inaccessible. This includes the facial expressions of the subjects, nonverbal or verbal communication, the sounds that were heard during the experiment (from the crowd or outside), or the focus of participants (e.g. gaze direction). The perspective of the people in the crowd is not taken and their subjective perception is not included in the study. As mentioned, this collaboration was formed after the experiments were conducted. Therefore, the questionnaire items used were also not appropriate for the questions that arose from the joint work. In particular, the questionnaire does not contain items on perceived unity in the crowd. However, we exploratively studied whether item responses differ between individuals who enacted different behavioural repertoires. Indeed they did, but the results are really not that informative beyond showing that individuals participating in repertoires such as racing, running and pushing were more aroused and had a somewhat less positive experience. Future work therefore needs to study how repertoires are appreciated and experienced by those inside the crowd.

Furthermore, a developmental perspective remains unanswered in this paper: where do the behavioural repertoires come from—are they resulting from emergent norms [60]? Subjects are in a situation that is very likely new to them. They are asked to move as a large group in an experiment as if they were going to a concert. Here, repertoires are certainly called up that are relevant to the instruction (concert entry) and that the subjects know from their own experience, or their cultural knowledge. For example, queuing is part of the cultural convention of entering a venue. However, other behavioural repertoires, in particular racing and overtaking seem to be less directly related to this setting. Here, participants seem to use a broader spectrum of movement repertoires. Racing, for example, seems to come from the context of sports competitions. In the experiments, an influence of the preceding steps (e.g. icebreaker experiment, queues when registering for the experiment, other experiments, even if these used different spatial setups) cannot be ruled out. Since the number of experimental runs studied is very limited, it is difficult to assess what are typical or less typical behavioural repertoires and where subjects actually spontaneously develop a new behavioural repertoire and how much improvisation there is.

Future research will have to verify the generalizability of the method, in three directions. First, different spatial layouts (e.g. bidirectional flow, intersections) will likely show different movement repertoires. Second, the method and its validity should be examined with data of real-life crowd situations (e.g. video recordings of festivals, train stations, evacuations). Third, although the sample was not limited to young students, and people over 60 were also well represented [53] a more diverse sample including for example underage children might show new behavioural repertoires.

## 5. Conclusion and future directions

The current paper has developed an observational method for describing crowd movement systematically. The method is to make an ethogram of all possible movement behaviours by focusing on those that, to human observers and actors, are experienced as socially meaningful and which, therefore, have consequences for how movement in the crowd is interpreted (both for observers and, we venture, for those inside). The method opens new theoretical perspectives on crowd movement: The ‘discovery’ of behavioural repertoires (such as queuing, racing, zipping, signalling another to go first, and so on) offers an understanding of how crowd members can perform highly complex behaviours both spontaneously and extremely fast, even when making rapid transitions between repertoires.

This new method and theoretical perspective together offer a range of applications for future research. The present work delivers the proof-of-concept for this, but to make this future application more viable it would help if the ethogram is developed further by applying it to many more crowd situations. Also, the ethogram is an exhaustive list of behaviours. For coding a large amount of data, this may need to be restricted and redefined such that codes are fewer and mutually exclusive. Such a simpler instrument is also easier to develop into an AI tool. Finally, in the future, behaviours should be coded continuously alongside trajectories, something which software tools such as PeTrack software [54] have recently made it possible to do.

Another future extension consists of delving deeper into how, inside the crowd, these repertoires are recognized, signalled and enacted. We believe the most tantalizing aspect of our findings is that they point to new ways of advancing crowd movement science, by integrating the engineering and physics perspectives with more interpretive social–psychological understandings. Our work suggests that we can do this by modelling and understanding crowds as highly dynamic environments in which people can move as individuals, as interactive units or large collectives, while behavioural repertoires structure the actions taken.

## Data Availability

The datasets supporting this article have been uploaded as part of the electronic supplemental material. The electronic supplementary material contains: (1) the four videos; (2) slides with the coded stills, both for raters and for the coding of social units and behavioural repertoires separately; (3) four coding tables (used for calculating the inter-rater reliability and for creating the alluvial charts). Electronic supplementary material is available on the Pedestrian Dynamics Data Archive: https://doi.org/10.34735/ped.2021.14.

## Footnotes

^1^
The exception was that in one of the runs which we used to develop the ethograms, a participant at the front of the crowd accidentally tripped while running towards the gate. The behaviour surrounding this fall (i.e. the people next to and behind her suddenly halting, checking on her while those who did not help remained spectators) was not seen in the four runs we ended up using for the final analysis and reporting in this paper. To us, this demonstrates that ethograms developed for particular social situations may never be completely exhaustive because when unexpected events happen, novel behaviours may occur.
